# Opportunities and challenges for global food safety in advancing circular policies and practices in agrifood systems

**DOI:** 10.1038/s41538-024-00286-7

**Published:** 2024-09-05

**Authors:** Andrew J. Pearson, Keya Mukherjee, Vittorio Fattori, Markus Lipp

**Affiliations:** 1 Agrifood Systems and Food Safety Division, Food and Agriculture Organization of the United Nations, Rome, Italy; 2 Tonkin + Taylor Ltd, Wellington, New Zealand

**Keywords:** Policy and public health in microbiology, Sustainability, Agriculture

## Abstract

Sustainable agrifood systems are needed to provide safe and nutritious food for the growing world’s population. To improve sustainability, transforming linear policies and practices in agrifood systems into circularity will be critical, with food safety considerations key for the success of this shift. This review provides a synthesis of the current and emerging risks, data gaps, and opportunities for food safety in agrifood initiatives aiming to advance circular economy models.

## Introduction

Working towards the Sustainable Development Goals (SDGs), the Food and Agriculture Organization of the United Nations (FAO) is committed to support the transformation to more efficient, inclusive, resilient, and sustainable agrifood systems^1^. Food safety is a key consideration in the transformation process. Food safety is linked with several critical aspects of agrifood systems. Firstly, ensuring food safety is fundamental to better health and improved nutrition. Over 600 million people worldwide fall ill every year from consuming contaminated food, with children under the age of five accounting for a third of all deaths from foodborne diseases^2^. Secondly, by preventing food contamination issues food safety enhances food security by minimising waste in the supply chain^1^. Thirdly, foodborne diseases exact an economic toll. In low- and middle-income countries the consequences of lost productivity from premature death and disability, as well as treating foodborne illness, mean unsafe food has a cost of about USD 110 billion a year^3^. Assurance of food safety facilitates international trade and is one of the foundations of economic development of agrifood systems worldwide^4^.

Global agrifood systems today face a poly crisis triggered by population growth, resource constraints, conflicts, climate change, biodiversity loss, food insecurity, and malnutrition^1^. Responding to these challenges requires re-thinking the existing economic model of agrifood systems to ensure that continued food security is balanced against resource efficiency, socioeconomic growth, and environmental sustainability^5^. Current policies and practices in agrifood systems typically operate along linear principles contingent on reliance on finite resources and high waste generation. For example, agriculture is reliant on high volumes of water, but water is used inefficiently, heavily abstracted from natural freshwater sources, in many cases above replenishing capacity, and post-use nutrient-rich wastewater is discharged into the environment^6^. Additionally, materials designed for single or limited use, with little potential for recovery, are used throughout the agrifood systems, including in food cultivation, collection, packaging, laboratory testing, and utensils for consuming food^7^.

A shift from linear practices in agrifood systems to circularity aims to do ‘more with less’ while reducing waste by closing material and processing loops. This change means value is derived in the reclamation and reuse of resources from waste and on the other hand products are redesigned to limit waste and enable upgrading and refurbishment to prolong their use. Further measures are taken to increase the efficiency of use and consumption to reduce the wastage in processing chains. Key outputs evaluated in the adoption of circular practices in agrifood systems tend to be sustainability in all dimensions - economic, environmental, and social^8^. However, adoption of circular practices is rarely preceded by the full understanding of the food safety hazards the changed approaches will present and the critical points that will see them emerge as risks^9^.

Circular practices in agrifood systems differ from those in other sectors, due to the perishable nature and short shelf-life of agrifood products. However, our current understanding and management of food safety is typically based upon the methods and systems used in a linear system. Considering these factors there is a need for detailed analyses of vulnerable points of entry and the types of potential risks within circular practices in agrifood systems to ensure the continued reliability of food safety risk management.

The established and emerging implications for food safety in circular practices were explored by undertaking a review across three themes where scientific and social initiatives on circularity offer promise in advancing sustainable agrifood systems (Table 1). The literature search strategy is reported in the Supplementary Information (Supplementary Note 1 and Supplementary Table 1). Studies were selected based on the hazards reported in circular feedstocks of interest that could transfer to food. This review also identifies common data gaps limiting food safety characterisation across the three themes and outlines a way forward on how to advance food safety in tandem with the adoption of circular practices.

**Table 1:** Summary of reviewed themes and initiatives for advancing circular practices and policies in the agrifood system with overview of risks and benefits to food safety

Theme	Water re-use	Food loss and waste	Packaging waste
Problem summary	Limited clean freshwater resources. Inefficiency of water use. Increasing water stress.	High avoidable food loss and waste at production, retail and household levels. Poor utilisation of food by-product waste	Finite virgin resources. Design for single use. High waste generation.
Initiatives considered in the review	Reuse in-system or in closed loops. Recycling alternative water sources.	Valorisation and conversion. Nutrient or energy recovery. Consumer education.	Reuse. Recycling materials. Redesign to reduce waste.
Risks to food safety	Persistence of pathogens, parasites and chemical contaminants. Anti-microbial resistance.	Persistence of pathogens, parasites and chemical contaminants. Anti-microbial resistance. Micro and nano-plastics. Compromised food handling.	Transfer of pathogens or contaminants from prior uses. Migration of non-intentionally added substances. Loss of integrity
Benefits to food safety	Phase out or segregation of persistent contaminants. Increased treatment efficacy.	Use of materials with reduced risk profiles. Phase out or segregation of persistent contaminants. Increased treatment efficacy. Improved education on household food safety.	Use of materials with reduced risk profiles. Improved integrity of packaging.

## Water re-use

Although water is a renewable resource, the demand for fit-for-purpose freshwater in the food production and processing sectors exceeds in many places the natural replenishment capacities of water sources^6^. Given the challenges of environmental impacts, stresses from the changing climate, and rising demand from a growing global population, sustainably managing water resources requires prioritising the allocation of freshwater, while limiting the impacts of overextraction. A range of initiatives are exploring the reuse and recycling of water in agrifood systems^10^. However, depending on the source of the reused or recycled water a number of food safety hazards can be present.

Pathogen presence, such as through contamination of water sources with faecal matter is a key quality concern for recycled water use (Table 2). Temperature variation and extreme weather events have also been linked to increased microbial loading of surface waters^11,12^. Irrigation of crops with pathogen-contaminated water is an established pathway for microbial contamination, a particular risk for crops eaten raw necessitating a high level of water quality^13,14^. Non-contact irrigation methods for recycled water, such as sub-surface drip, are reported to minimise the percentage transfer of pathogens to edible components and reduce the water quality requirements^15^. A study of aquaponics system confirmed that Shiga toxin-producing *Escherichia coli* was present in the recirculating water and could contaminate root surfaces of vegetables, although it was not reported to internalise into leaves or fruit^16^ An emerging concern is the potential for root uptake of viruses (norovirus and hepatitis A virus) from soil or in hydroponic systems using inoculated water^17,18^. Even without evident human or animal waste sources, risks remain, for example, pathogens have been reported in produce wash waters and cleaning waters which persist in closed loop systems, particularly when treatment facilities are inadequate (Table 2^19,20^;). In crops and produce that are to be further processed or cooked the microbial risks are lower and the treatment requirements for irrigation water are less^14^. Similarly, lower quality water may be fit-for-purpose for recycling into non-contact uses, such as in heat transfer systems^21^.

**Table 2:** Examples of hazards reported in water sources with potential to be recycled

Water source	Reported hazards		Source
	Microbiological	Chemical & physical	
Food production wash and process water	Bacteria	Sanitisers	^19^
Industrial /extractive process water		Heavy metals PFAS Dyes	^85,86^
Agricultural run-off	Bacteria Protozoa	Pesticides Veterinary medicines	^87–89^
Greywater	Bacteria Viruses Protozoa	Narcotics Personal care products	^90,91^
Stormwater	Viruses Bacteria Protozoa	Heavy metals Surfactants Plasticizers Pesticides Hydrocarbons and PAHs Tyre wear particles	^92,93^
Treated wastewater	Viruses Bacteria Antimicrobial resistance genes Protozoa Helminths	Pharmaceuticals Narcotics Personal care products Cleaner/sanitisers Microplastics PFAS	^94–98^

There are limited supporting studies on the microbial risks present in recycled water used for stock water. Australian guidance focuses on helminths as a key risk, given the potential for these to transmit to livestock and then be a food safety risk through eating undercooked meat, for example, the risk from *Taenia solium* in pigs^22^. A Californian advisory panel on disinfected tertiary recycled water for non-dairy stock water concluded there was insufficient evidence to determine if the use would present a risk to public health, primarily from the uncertainty related to pathogens^23^.

The specific risk to food safety is dependent on the potential for the hazard to persist in the water source, or on contacted surfaces and soil, and subsequent transfer to livestock or crops. Assessment of recycled water drawn from different sources indicates a range of possible chemical hazards for food safety are present (Table 2). Studies have reported a number of chemicals that accumulate in produce from irrigated soil^24–26^. For example, the anti-convulsant drug carbamazepine has been reported in field trials to concentrate from wastewater effluent treated soil into wheat grain, tomato, and lettuce^27^. An emerging concern is the contamination of agricultural soils with per and polyfluorinated alkyl substances (PFAS) due to irrigation with reused or treated wastewater^28^.

The possible presence of both human pathogens and levels of pharmaceuticals or biocides in recycled or reused water presents a concern area for resistant organisms. Studies of recycled water sources have shown broad prevalence of antibiotic resistance genes (ARGs), for example, tertiary treated wastewater as well as vegetable processing water containing resistance genes for multiple antibiotic families^29^.

Acknowledging the importance of characterizing and validating the safety and quality of water the Joint FAO & WHO Expert Meeting on Microbiological Risk Assessment has developed technical reports on the use and reuse of water in different agrifood sectors^14,20,21,30^.

## Food Loss and Waste (FLW)

Between the post-harvest and retail stages of the food chain alone, about 14% of food produced globally is lost, while around 17% of total food produced is wasted at the retail and consumer stages^31,32^. Stemming FLW is an important component of increasing the sustainability of agrifood systems and their guiding principles and actions that promote various circular initiatives^33^. Aside from supply chain measures to reduce and redistribute FLW, it can also be valorised, for example through the extraction of bioactives or conversion to new food or feed sources, or have its nutrient content recovered through processes such as composting and application to productive land^34^. A concern is that contaminants in sources of FLW have the potential to persist and accumulate through these circular practices, presenting food safety risks when the recovered product re-enters the food chain. Surveys of food waste have reported the common presence of foodborne pathogens including *Listeria monocytogenes, Salmonella* spp. and *Yersinia* spp.^35^. Transmission of parasites could also be a concern if food waste valorised for feed purposes contains infected tissue, such as encysted *Trichinella* spp. in wild or domestic pork^36^. Lastly, ARGs have been found to occur in sources of FLW and increase significantly with storage time; animal product FLW is a particular risk given a higher occurrence of ARGs coupled with the presence of pathogens^37,38^.

The persistence of pathogens through the processing of food waste is commonly technology or treatment dependant. Correct maturation of food waste composts will sufficiently reduce pathogen loadings^39^, and many countries have statutory or voluntary standards for time and temperature requirements for compost sanitization^40^. The abundance of ARGs decreases in the stabilization phase during most composting operations as the host bacteria are reduced^41^.

Transfer of prions represents an area of potential concern where food waste may be valorized into animal feeds as the level of treatment to denature these proteins may need to exceed that for pathogen deactivation^42^. Screening for Scrapie prion elimination in food waste cultured yeast, used as a feedstock for animal feed, found reduction in levels occurred, although potentially not to the degree where it could be the sole control^43^.

Contamination with various fungal species is one of the major factors that lead to FLW. As a consequence, food waste presents a source of mycotoxins that can persist if valorised into food or feed uses. A review of mycotoxins in food by-products identified aflatoxin B1, ochratoxin A, fumonisins, deoxynivalenol and zearalenone as commonly found^44^. By-products of the vegetable oil industry, including flours and groats had concentrations of deoxynivalenol upto 980 µg/kg, aflatoxins upto 1.5 µg/kg and zearalenone upto 79 µg/kg^45^.

Digestates from biogas production, which included food wastes, were reported to contain a range of emerging or persistent organic pollutants (POPs), including dioxins, polybrominated diphenyl ethers (PBDEs), polycyclic aromatic hydrocarbons (PAHs), naphthalene, PFAS, phthalates, and nonyl phenol^46^. Furthermore, biofertilizers from food waste digestates were found to have an array of substances, including high concentrations of nicotine, caffeine, fungicides, parabens and pharmaceuticals^47^.

Household food waste composts have also been reported to have residual pesticide presence^48^, while a study of cadmium, arsenic, nickel, copper, and mercury ranges across different foods reported many could exceed regulatory limits for composts, should FLW be directed to this route^49^. Valorisation of bio-actives from waste fruit peels and rind presents a pathway for surface-bound pesticide residue to concentrate^50^, while residual pomace, seeds, and extracts also concentrate certain residues^51^.

A developing area for FLW valorisation is as a substrate for raising insect species. For example, there are opportunities to rear insects on heavily mycotoxin-contaminated crops that would not be acceptable for food or feed^52^, with some evidence that insects thrive on contaminated feed and do not accumulate the toxins^53^. It is cautioned, however, that the understanding of detoxification pathways in insects is uncertain and there is a potential risk for masked-mycotoxins or toxic metabolites to be present. Furthermore, insects also accumulate other toxicants such as heavy metals, pharmaceuticals, and mineral oils from food waste^54^. Raising insects on organic waste streams also presents a potential risk from pathogens, with *Salmonella* spp. and *Bacillus cereus* detected in black soldier fly larvae and their environments after rearing^55,56^. An emerging concern is that insects can also carry bacteria with transferable ARGs^57,58^.

Commonly food waste is contaminated with packaging if it cannot be separated or is improperly screened^35^ presenting a risk of migration of packaging contaminants and fragmentation to micro- and nano-plastics^7^. Composts contaminated with plastic, as established through the presence of microplastics, had high concentrations of plastic additives such as di(2-ethylhexyl) phthalate (DEHP) and nonanal, which transferred into agricultural soils^59^. Occurrence data for nano-plastics is currently constrained by the analytical methods and it is unclear whether their exposure through the diet has negative impacts on human health. However, nano plastics are reported to transfer into crops, as a result presence in food waste composts would present a source into agrifood systems^60^.

Circular practices for FLW present an opportunity to reduce the dietary contaminant burden if they replace other food or feed sources with comparatively elevated contaminant levels^61^. For example, valorisation of food waste into animal feed to substitute for feed sources, such as fishmeal, that contain contaminants such as mercury or POPs^61^. In a study of pelletized food waste intended for animal feeds, mycotoxins, and pesticides were below detection levels, while lead, cadmium, and arsenic complied with regulatory limits^62^.

Increasing awareness of sustainability issues is also resulting in consumers increasingly making choices considering the environmental aspects of purchased foods in addition to safety and nutrition^1,63^. Household food waste, much of which is avoidable can present a high proportion of total food waste volumes, so supporting this shift in behaviour will be critical to enact circular systems^64^. Adoption of certain behaviours to support circularity at home, however, potentially runs in conflict with advised food safety practices by introducing risky actions. As sustainability becomes increasingly important for consumers, it will be important to monitor and prevent fraudulent practice^65^. Food fraud related to FLW could include adulteration with unsuitable or hazardous feedstocks, or the provision of misleading information as to the use of sustainable practices^66^. While the former can lead to potential food safety risks in the consumed product, the latter can derail efforts to shift to more sustainable food practices and erode consumer trust.

A measure to limit food wastage is to avoid throwing out food that is still fit for consumption. However, this presents a challenge for food safety as consumers often have a poor understanding of when the expiry of shelf-life is a food safety concern or solely a quality issue^67^. Attempts to limit home food waste may cause risky behaviours such as consuming spoiled foods, saving leftovers incorrectly or for too long, or consuming rinds, peels, and other low-edibility parts of food to limit these going to waste^68^. If guidance is not available consumers commonly draw on unproven or unsafe techniques to support reducing food waste, for example relying on smell or taste to determine safety, or cutting off mouldy parts of food despite the risk that mycotoxin can be diffused into areas without visible contamination^69,70^.

## Packaging waste

Packaging plays a key role in food safety and food security, through protecting food from contamination and, thus, reducing food waste. However, the high waste burden from current packaging materials necessitates innovation to improve sustainability^71^. Among other food packaging materials (metals, glass, plastics, paper, cardboard, etc.), plastics account for significant food packaging-related waste due to large production volumes, much of which are of non-biodegradable chemistry. An estimated 37.3 Mt of plastics was used globally in food packaging in 2019^7^.

Recycling is seen as key to improving the circularity of food-related plastics. However, recycled plastics contain a broad range of non-intentionally added substances (NIAS) that present as potential contamination risks for food packaging uses and a challenge to identify and risk assess (Table 3^72^;). NIAS commonly results from the retention of plastic additives from recycling feedstock and the inability to segregate non-food plastics. There are also limits to the recyclability of certain materials, for example, polypropylene shows degradation through reprocessing^73^, which restricts future applications of recycled plastics, or leads to them having unpredictable performance or NIAS migration risks.

**Table 3:** Summary of non-intentionally added substances reported in recycled plastics

Material	Non-intentionally added substances	Reference
Recycled HDPE and LDPE pellets	Volatile and semi-volatile organic substances, including surfactants, antioxidants, and odours from decayed food. PBDEs and benzotriazole UV stabilizers	^99,100^
Recycled pellets and flakes for various recycled plastic types	Organophosphate esters	^101^
PET	Ethanol and ethylene glycol; and flavourings such as anethole and limonene	^102^
Marine and riverine recovered plastics	PAHs, PBDEs, and PCBs	^103^

Designing packaging for reuse offers the ability to extend the lifecycle of plastics or other food contact materials and reduce the amount of waste. Successful reuse relies on avoiding cross-contamination between uses and ensuring the product retains integrity. Adequate cleaning is critical as pathogens are reported to survive in packaging reuse, for example, polypropylene used in reusable produce crates was reported to have a higher risk of *Salmonella spp*. cross-contamination in cauliflower than single-use materials^74^. Proper decontamination of any chemical contaminants before reuse is also important, particularly where the prior user has stored non-food materials. Misuse (storage of alcohol, cleaning products, and fuels) of refillable PET bottles was identified as a potential source for organoleptic impacts in mineral water and soft drinks^75^. A greater concern is that unsafe storage of hazardous chemicals in reusable or refillable containers could lead to toxicity if not decontaminated.

Degradation is a risk with long-term reuse of packaging causing the migration of contaminants in the container as well as release of physical fragments, such as microplastics. A study of polyethylene bottles reused for one year indicated a range of different migrated substances, including plasticisers, antioxidants, and photoinitiators, with migration rates enhanced following dishwashing^76^.

Food waste-sourced polymers, as a biodegradable replacement to plastics, are subject to potential concerns over contaminant loading of food waste and the potential for these to cycle into the food packaging^77^. Hazards could include myco- and phyto-toxins, POPs, heavy metals, process contaminants such as acrylamide that are formed in cooked foods, as well as allergenic proteins from plant materials^77^. As packaging waste circularity becomes a draw for consumer decision making there can be economic incentive for fraud in the use of unsuitable materials. Recent examples of food contact materials marketed as biobased resulted in recalls due to the use of, and migration risk from, melamine^78^.

Packaging plays an important role in managing food waste, consequently, there are opportunities for innovation streams to focus on improving shelf life, losses in storage/transport, minimizing contamination, and helping retailers/consumers better identify spoilage. However, utilizing plastics-based packaging to reduce food waste often necessitates a trade-off against packaging reusability or recyclability. For example, multilayer packaging has applications that increase the longevity of food while also providing a barrier to the migration of NIAS. However, such materials are at present difficult to recycle^79^. Adding packaging material, such as incorporating measuring tools or seals, or packaging smaller quantities of food, may also be justified if it decreases food waste by supporting consumers to better use or store the food^80^.

## A way forward: Challenges and opportunities

Adopting circular economy models is a step many countries have begun, or will need to begin, to manage the sustainability of the agrifood sector and maintain food security. Transformation to models that utilise waste products, be they wastewater, food waste, organic material, or packaging presents risks due to the safety hazards these may contain, and/or generate. Contemporary food safety has developed in association with the linear processes of agrifood systems. Consequently, food safety also must adapt to characterise circular practices, from which existent and emerging hazards can generate risk.

While precaution should not hinder efforts to advance circularity in agrifood systems, the failure to suitably address risks that increase the foodborne disease burden will likely be detrimental to progress, as producers, consumers, and trading partners lose trust in the produced food. Addressing food safety as a core component in innovation and adoption of circular practices will ensure that risks are characterised, and risk management controls are validated concurrently with the change.

Advancing circular policies and practices, in particular repurposing what is waste in a linear model to a feedstock resource, changes the pathways by which food safety hazards lead to risk. Transforming agrifood systems to increase circularity must contend with and address many uncertainties in relation to impacts on food safety. Innovations in adopting circularity into agrifood systems have often been supported with limited research into food safety^77^ and there has been little focus on hazards that can emerge and accumulate in adopting circular practices^9^. Table 4 identifies data gaps that limit robust risk assessment and risk management of food safety in a circular agrifood system.

**Table 4:** Identified data gaps for food safety risk in adopting circular agrifood policies and practices

*Microbiological*	*Chemical*	*Other*
Survival and internalisation of pathogens into crops from environmental applications.	Identification and characterization of NIAS in packaging materials and migration to food in reuse	Risks related to ARGs, particularly where antimicrobials co-occur in the feedstock
Potential exposure to emerging or opportunistic pathogens.	Emerging contaminant hazard characterisation and quantifying uptake into food crops and species	Characterizing the hazard of micro- and nano-plastics
Survival of pathogens through valorisation, such as in insect feed.	Natural toxin persistence and transformation in circular systems.	Vulnerability to food fraud from use of unsuitable feedstocks, or misrepresenting circular processes.
Significance of parasites	Allergenic potential of biopolymers as well as retention of allergens through circular systems	
Efficacy of decontamination techniques		

**Fig. 1:**
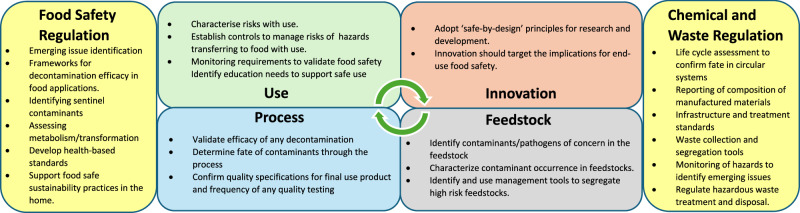
Focus points for food safety in transforming to circular practices and policies in agrifood systems. To successfully advance circular policies and practices all agrifood system actors have a role to play in ensuring sustainability in all its dimensions, while also focusing on producing safe food. Adopting a “safe-by-design” approach in innovating ensures that food safety risks are characterized throughout the research and adoption of circular processes. Food safety hazards in circular agrifood systems can be present in feedstock, processed material, and final products and these hazards have varying implications for food safety. Characterization of the risk to consumers from these hazards, and if required development and validation of risk management, enables a proactive approach to maintaining food safety. Food safety regulation must enable and support agrifood systems transformation and must also align with chemical approval and waste legislation to support life cycle assessment and good management of waste infrastructure and treatment.

Ensuring that food safety hazards are characterised throughout researching and adopting circular policies and practices underpins the assurance that the resulting food is safe when produced or packaged using these new approaches. Figure 1 illustrates the roles for researchers, farmers, food manufacturers, regulators, and consumers in supporting the transformation to a safe circular agrifood system. Sustainability initiatives and national research funding should ensure food safety is considered by all actors in the agrifood system.

Although this publication places the onus on characterising food safety risks, there are also opportunities in developing and adopting circular policies and practices to improve food safety outcomes. Either directly through the introduction of new materials with reduced risk profiles or treatment processes that produce a safer food product, or indirectly through the economic or societal pressure to phase-out contaminants that persist in circular systems.

Ensuring clear guidelines are established to assess and manage food safety risks and that regulations adequately cover the new uses of waste materials in agrifood systems ensure a proactive approach to maintaining food safety while advancing circularity. Agricultural and food safety legislation also must interface well with chemical and waste legislation to support lifecycle assessment and management of waste treatment infrastructure. An emphasis could be placed on reviewing food safety regulatory approaches with the aim to limit food wastage where eventuation of a risk is unlikely^33^. Operating within and harmonising with Codex Alimentarius standards ensures a level and transparent approach to food safety regulation and consequently limits wastage in trade^33^.

Outcomes focusing on one aspect of waste, e.g., reduction in packaging waste may have unforeseen impacts on increasing food waste if solutions do not maintain integrity and result in quicker spoilage^81^. There are opportunities for initiatives that increase waste in one area, such as changing packaging attributes, to have a net benefit on waste if they support informed decision-making for avoiding purchasing or using too much food, or identifying when food is spoiled or unsafe in retail and in-home storage^82^.

With the reuse of waste, it is possible that consumers may have a disgust factor linked to consuming foods from circular agrifood practices^83^. To avoid food loss, transparency in how any food is produced is important, particularly if environmental sustainability is important for consumer choices. Overcoming disgust further reinforces the need for strong food safety messaging to provide assurance that health is not at risk^84^.

To summarise, various pressures are placed on agrifood systems by the way we currently produce, distribute, and consume food. This pressure is driving global change to be more efficient, inclusive, resilient, and sustainable while realizing multiple United Nations Sustainable Development Goals^6^. To ensure sustainable agrifood systems that deliver safe, affordable, and healthy diets, against these pressures requires concrete actions. Microbiological, chemical, and physical contaminants are known to persist in certain waste streams and by-products, which through reuse could exacerbate existing food safety issues or cause new risks to emerge. Failure to address food safety in adopting circular systems may lead to an increased disease burden and set back these initiatives.

Going forward innovation can be supported by focusing research efforts on developing and adopting circular policies and practices in agrifood systems, on the impacts to food safety, and on exploring opportunities for improving food safety outcomes. Research and industry initiatives can be supported with clear regulations which can be reviewed as to the flexibility to reduce waste whilst protecting food safety. Transformation to circular agrifood systems offers remarkable promise in achieving goals focused on sustainable, inclusive, and efficient development. All levels of the food chain from farmer to consumer, as well as regulators, have a role to play in protecting food safety while advancing circularity in agrifood systems.

## Supplementary information

Supplementary Information
