# Supplementary material for: Opportunities and challenges for global food safety in advancing circular policies and practices in agrifood systems
Source: NPJ Sci Food. 2024 Sep 5;8:60. doi: 10.1038/s41538-024-00286-7 (PMC11377707; doi:10.1038/s41538-024-00286-7)
Supplement: Supplementary file 1 — Supplementary Information [file 41538_2024_286_MOESM1_ESM.pdf]

Supplementary Information

Supplementary Note 1: Search strategy for exploring established and emerging implications for food safety in circular practices.

Peer reviewed scientific studies, relevant international and national regulatory guidance, and technical reports were obtained from Google Scholar and PubMed using a combination of keywords for each theme (Supplementary Table 1). The search was not limited by publication year or other classifications, however recent publications tended to be better representative of circular practices and associated food safety hazards. Full papers were reviewed based on the relevance of the title and abstract content. International regulatory guidance was primarily drawn from the World Health Organization and Food and Agriculture Organization of the United Nations resources.

Supplementary Table 1: Search strategy for exploring established and emerging implications for food safety in circular practices.

**Theme**	**Water re-use**	**Food loss and waste**	**Packaging waste**
Theme terms	“Water re-use” OR “Recycled water” OR “Treated Wastewater” OR “Tertiary treated water” OR “Stormwater” OR “Process water” OR “Greywater” OR “Run-off”	"Food waste” OR “Food loss and waste” OR “By-product”	“Resue” OR “Recycling” OR “Redesign” OR “Biopolymer” OR “Biodegradable”
Application terms	“Irrigation” OR “Aquaculture” OR “Agriculture” OR “Livestock” OR “Farming” OR “Food production” OR “Circular agrifood” OR “Circular agriculture” OR “Hydroponics” OR “Aquaponics” OR “Controlled Environment” OR “Cooking” OR “Catering” OR “Consumer”	“Aquaculture” OR “Agriculture” OR “Livestock” OR “Animal raising” OR “Insects “OR “Feed” OR “Farming” OR “Food production” OR “Circular agrifood” OR “Circular agriculture” OR “Bioactive” OR “Valorisation” OR “Upcycling” OR “Compost” OR “Digestate” OR “Nutrient Recovery” OR “Biofertilizer” OR “Consumer”	“Packaging” OR “Fibreboard” OR “Plastic” OR “Cardboard” OR “Bottle” OR “Container” OR “Biopolymer”
Hazard terms	“Contaminant” OR “Hazard” OR “Pathogen” OR “Food Safety” OR “Chemical” OR “Toxin” OR “Toxicant” OR “Foodborne Illness” OR “Virus” OR “Parasite” OR “Helminth” OR "Antibiotic resistance”	“Contaminant” OR “Hazard” OR “Pathogen” OR “Food Safety” OR “Chemical” OR “Toxin” OR “Toxicant” OR “Foodborne Illness” OR “Virus” OR “Parasite” OR “Helminth” OR “Prion” OR “Microplastic” OR "Antibiotic resistance”	“Contaminant” OR “Hazard” OR “Pathogen” OR “Food Safety” OR “Chemical” OR “Toxin” OR “Toxicant” OR “Migration” OR “Additive” OR “Leaching” OR “Degradation” OR “Microplastic” OR “Taint” OR “Allergen”
